# 2PP-Hydrogel Covered Electrodes to Compensate for Media Effects in the Determination of Biomass in a Capillary Wave Micro Bioreactor

**DOI:** 10.3390/bios14090438

**Published:** 2024-09-09

**Authors:** Sven Meinen, Steffen Brinkmann, Kevin Viebrock, Bassant Elbardisy, Henning Menzel, Rainer Krull, Andreas Dietzel

**Affiliations:** 1 Institute of Microtechnology, Technische Universität Braunschweig, Alte Salzdahlumer Str. 203, 38124 Braunschweig, Germany; 2 Center of Pharmaceutical Engineering (PVZ), Technische Universität Braunschweig, Franz-Liszt-Str. 35a, 38106 Braunschweig, Germany; 3 Institute of Biochemical Engineering, Technische Universität Braunschweig, Rebenring 56, 38106 Braunschweig, Germany; 4 Institute of Technical Chemistry, Technische Universität Braunschweig, Hagenring 30, 38106 Braunschweig, Germany

**Keywords:** microbioreactor, biomass sensing, two-photon polymerization, 3D-printed hydrogel

## Abstract

Microbioreactors increase information output in biopharmaceutical screening applications because they can be operated in parallel without consuming large quantities of the pharmaceutical formulations being tested. A capillary wave microbioreactor (cwMBR) has recently been reported, allowing cost-efficient parallelization in an array that can be activated for mixing as a whole. Although impedance spectroscopy can directly distinguish between dead and viable cells, the monitoring of cells in suspension within bioreactors is challenging because the signal is influenced by the potentially varying properties of the culture medium. In order to address this challenge, an impedance sensor consisting of two sets of microelectrodes in a cwMBR is presented. Only one set of electrodes was covered by a two-photon cross-linked hydrogel to become insensitive to the influence of cells while remaining sensitive to the culture medium. With this impedance sensor, the biomass of *Saccharomyces cerevisiae* could be measured in a range from 1 to 20 g L^−1^. In addition, the sensor can compensate for a change in the conductivity of the suspension of 5 to 15 mS cm^−1^. Moreover, the two-photon cross-linking of hydroxyethyl starch methacrylate hydrogel, which has been studied in detail, recommends itself for even much broader sensing applications in miniaturized bioreactors and biosensors.

## 1. Introduction

**Figure 1:**
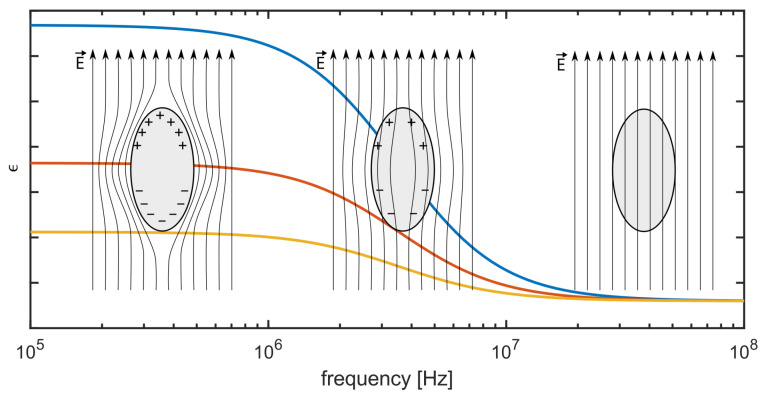
The general course of the real part of the relative permittivity ϵ as function of frequency for increasing concentrations (from yellow to red to blue) of living cells in suspension (based on [13]). A higher permittivity change is obtained at lower frequencies, whereas the effect disappears at higher frequencies and cells do no longer contribute to permittivity. The three schematics illustrate the respective degrees of cell membrane polarization and the interaction with the electric field $\vec{E}$.

In recent years, it has become of high relevance to miniaturize bioreactors to use them as powerful tools for biopharmaceutical screening applications [1,2,3,4]. Microbioreactors (MBRs) are used for screeninig appliactions of microorganisms due to the low consumption of precious formulations of active pharmaceutical ingredients and due to the feasibility of parallelization [5,6]. A new kind of MBR was developed by [7,8], which was named capillary wave microbioreactor (cwMBR). The cwMBR was manufactured, using the laser direct writing of photosensitive glass followed by chemical etching and had a volume of 8 μL. The fluid was mixed by vertical oscillation at resonance frequencies, which led to high mixing rates and therefore high oxygen transfer coefficients ($k_{L}a$) of up to 340 h^−1^. Using an optical density sensor setup, exponential growth rates of *Escherichia coli* B21 were tracked in the cwMBR, thereby proving the applicability of the system in biological screening applications. However, the oscillating sensor setup was prone to disturbances, e.g., small variations in aligning the glass fibers for read out. Moreover, a method is preferred that also can distinguish between living and dead cells [8]. Based on the cell membrane polarization, biomass becomes accessible when using electrical impedance spectroscopy (EIS). This polarization disappears at specific frequencies depending on the cell properties and cell size and only occurs on living cells [9,10,11,12]. How the polarization decreases with the frequency of the electric field and influences the permittivity is illustrated by Figure 1. At high frequencies, the cell conductivity is higher because the current can pass the membrane and travel through the ion-rich intracellular environment, whereas the current must flow around the cell at lower frequencies. This effect is a consequence of charge relaxation and is known as β-dispersion.

The real part of the permittivity ϵ, representing the capacity of the system, assuming an ideal Debye relaxation as a function of the frequency *f*, is shown in (1). Here, $\epsilon_{l}$ is the permittivity at lower frequencies, at the top of the step in Figure 1 and $\epsilon_{h}$ is the permittivity at higher frequencies when the β-dispersion has reached the lowest level [14].

(1) $$\epsilon =\epsilon_{h}+\frac{\epsilon_{h}-\epsilon_{l}}{1+{\left(2\pi f\;\cdot \;\tau \right)}^{2}}$$

The relaxation time τ depends on various cell parameters such as the size, shape and membrane capacity. Larger cells exhibit a longer relaxation time. At the characteristic frequency, $f_{c}=\frac{1}{2\pi \;\cdot \;\tau }$, the dispersion step is half through. In measurements, $f_{c}$ can therefore be used to determine cell properties, mainly the size. The difference $\Delta \epsilon =\epsilon_{l}-\epsilon_{h}$ is proportional to the viable cell concentration [15,16]. In experiments, this change is revealed by the change in capacity *C* measured at frequencies below and above the dispersion.

Monitoring biomass by electrical impedance spectroscopy in batch cultivation is challenging for low cell concentrations in particular with the influence of the electrolyte, which superimposes the impedance signal generated by the cells [17]. During cultivation, the composition and thus the conductivity also changes due to various metabolic processes, such as the formation of alcohol and the breakdown of organic acids. For cultivations with only minor changes in the medium, it is possible to subtract the once-measured impedance of the medium. Moreover, it was proposed that the capacity measured at two different frequencies could be used to fit an exponential function to the data and subtract it to compensate for the electrode polarization, which also interferes with the measurement of biomass [18]. However, in a microbioreactor (MBR) intended for biopharmaceutical screening, the influence of pharmaceutical formulations in the culture medium is too strong to be compensated for in this way. In order to completely suppress the influence of the culture medium and of electrode polarization, a reference electrode system (RES) covered by a semi-permeable membrane was introduced in addition to the measurement electrode system (MES) [19]. This membrane was a hydrogel, which allowed the culture medium to pass but stopped the cells to enter the electric field between the electrodes of the RES.

Hydrogels are a group of materials which, due to their distinctive properties, are increasingly used in life sciences, in particular biotechnology and tissue engineering [20,21,22,23]. These properties include mechanical characteristics, similar to those of natural tissue, and very good biochemical compatibility. Both make hydrogels favored materials for working with living cells or whole cell cultures and investigating their interaction with microenvironments [24,25,26,27]. A hydrogel is a polymer network that can absorb large amounts of water in the size range of multiples of its own dry mass [28]. Due to this process of swelling, the network is stretched and, depending on its internal pore structure, it is permeable to the diffusion of electrolytes but forms a barrier to suspended particles, including cells [21]. A suitable method for the fabrication of arbitrarily complex 3D hydrogel structures with a spatial resolution of a few micrometers is given by two-photon polymerization (2PP) [29,30,31]. In 2PP, a photosensitive precursor solution becomes cross-linked within the two-photon absorbance volume of a focused ultrashort laser pulse. Complex, three-dimensional structures are produced by the movement of the focal volume through the photosensitive solution [32].

Hydroxyethyl starch methacrylate (HES-MA), cross-linked with UV light, using the water-soluble photoinitiator lithium phenyl-2,4,6-trimethylbenzoylphosphinate (LAP), has been proven to be biocompatible [33,34,35] and was investigated for the use as drug delivery system [36]. Up to now, the cross-linking of HES-MA and LAP using 2PP and its suitability for use in RES on angled sidewalls of a MBR has not yet been investigated.

This paper presents an integrated biomass impedance sensor that makes it possible to compensate for the effects of the medium on the biomass signal. To this end, a new manufacturing option is presented for both the cwMBR and the impedance sensors. One focus of the work is the generation of hydrogel-based 3D structures using 2PP to be used as part of the biomass sensor.

## 2. Materials and Methods

### 2.1. Fabrication of the cwMBR

In previous works, the cwMBRwas manufactured using photosensitive Foturan^®^ (Schott AG, Mainz, Germany) glass and a laser direct writing process [7]. This technology had some advantages, such as the capability of manufacturing truly 3D strucures in a monolithic way and adjusting the shape by simply changing the computer assisted design. However, the process had a drawback. It was expensive regarding the material and regarding the fs-laser microstructuring process time. Therefore, laser ablation and glass molding were considered as two different alternatives. The resulting surface roughness $S_{a}$ can be critical for cell cultivation and was measured by means of a laser scanning microscope (VK-X260K, Keyence, Osaka, Japan).

For the glass molding, a positive master was fabricated using laser direct writing in Foturan^®^, taking isotropic shrinkage of 26% [37] into account. This master was placed in a mechanically milled polytetrafluorethylen form which was filled with liquid polydimethylsiloxane (PDMS, Sylgard 184, Merck KGaA, Darmstadt, Germany). After curing at room temperature, a negative PDMS master was obtained into which a mixture of Glassomer L50, which is a hydrogel packed with fused silica particles [37], and 1 wt% hardener (both Glassomer GmbH, Freiburg im Breisgau, Germany) was poured. After curing under UV light with a dose of 2.25 J cm^−2^, the debinding in a muffle furnace (Laborofen VMK—135 S, Linn High Therm GmbH, Eschenfelden, Germany) at 600 °C with oxygen supply and the sintering in an oven at 1300 °C (Muffelofen LT 9/14, Nabertherm GmbH, Lilienthal, Germany) were executed following the recipe of the vendor.

For the laser ablation route of cwMBR manufacturing, 1.1 mm and 0.5 mm thick borosilicate glass 4-inch wafers (Borofloat^®^ 33, Schott, Mainz, Germany) were used. The process including ablation, bonding and thermal annealing was described earlier [38]. In the thicker wafer, through-holes were ablated. A wall angle of 50° was obtained by reducing the size of the ablation from layer to layer toward the bottom. The thick wafer was thermally bonded at 650 °C to the unstructured thinner wafer, establishing an optically clear bottom for each cwMBR; the entire wafer stack was annealed at 760 °C to gain smooth side walls.

### 2.2. Fabrication of the Impedance Sensor

**Figure 2:**
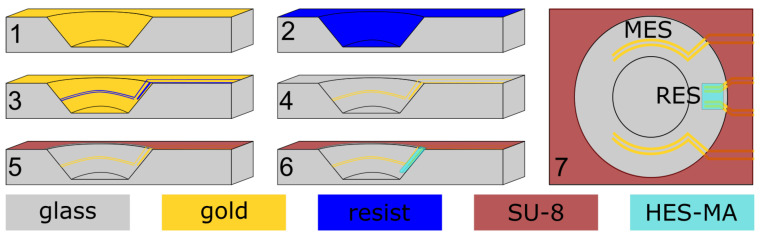
Schematic illustration of impedance sensor microfabrication. 1: sputtering of a chromium-gold double layer. 2: electrodeposition of resist. 3: UV exposure and resist developing. 4: metal etching. 5: spin coating of SU-8, exposure and developing. 6: structuring the hydrogel HES-MA using 2PP. 7: top view of design with measurment electrode system (MES) and reference electrode system (RES).

The impedance sensor electrodes were manufactured within the cwMBR as illustrated in Figure 2.

Layers of chromium (resulting in 8 nm on planar surface) and gold (resulting in 250 nm on planar surface) were sputtered (LS 440 S, Ardenne Anlagentechnik GmbH, Dresden, Germany) onto the finished cwMBRs before the resist (Intervia 3D-N, Rohm and Haas, Philadelphia, PA, USA) was electrodeposited at a voltage of 105 V at 26 °C and dried under vacuum for 3 h. The resist was then exposed in two steps. First, the electrodes inside the cwMBRs on angled sidewalls were exposed using a laser microstructuring workstation (microSTRUCT C, 3D Micromac AG, Chemnitz, Germany) equipped with a femtosecond laser (Pharos, Light Conversion, Vilnius, Lithuania) at 343 nm with a power of 1 mW at a writing speed of 24 mm s^−1^. As a next step, the patterns of electric supply paths with contact pads were exposed on the planar surface using a mask aligner (EVG^®^ 620, EV Group, St. Florian am Inn, Germany) at a dose of 0.7 J cm^−2^. The resist was developed (InterVia 3D-N Developer, Rohm and Haas, Philadelphia, PA, USA) before the metallic coating was etched in aqueous iodine–potassium iodide solution and subsequently in an alkaline chromium-etching solution. Next, the remaining photoresist was stripped with acetone. For passivation of the electrical leads, SU-8 (SU-8 2050, MicroChem Corporation, Newton, MA, USA) was spin-coated on the wafer. Inhomogenities of resist thickness caused by the cwMBRs recesses did not disturb lithographic patterning according to the manufacturer’s specifications. Subsequently, the wafer was divided into individual cwMBR systems, each measuring 10 mm × 60 mm, using a dicing saw (DAD 320, Disco, Tokyo, Japan). Following this, the hydrogel was printed into the separated cwMBRs, as detailed in Appendix A.1.

### 2.3. Yeast Cultivation

*Saccharomyces cerevisiae* LBGH1022 was applied as a model organism for the determination of biomass concentration by EIS. Therefore, different biomass concentrations were achieved by the centrifugation of a *S. cerevisiae* culture and resuspension in different volumes of culture medium supernatant. First of all, a pre-culture of cryopreserved cells was incubated overnight in 50 mL yeast extract peptone dextrose (YPD) medium containing 10 g L^−1^ yeast extract (Carl Roth, Karlsruhe, Germany), 200 g L^−1^ peptone (Bacto^TM^ Peptone, Thermo Fisher Scientific, Waltham, MA, USA) and 20 g L^−1^ glucose (Carl Roth, Karlsruhe, Germany) in deionized water. Incubation was performed in 500 mL shake flasks with baffles at 30 °C and a shaking frequency of 20 min^−1^ with a shaking diameter of 50 mm (ISF1-X incubator, Adolf Kühner AG, Birsfelden, Switzerland). The main culture was inoculated with an optical density of 0.1 and incubated at identical conditions like the pre-culture for 16 h. After reaching a final optical density of 8.9 (Libra S11, Biochrom Ltd., Cambridge, UK), biomass standards between 1 and 23.6 g L^−1^ bio dry mass concentration were produced by centrifugation at 200× *g* for 10 min and following resuspension in supernatant of the culture. In comparison to the impedance sensor, the bio dry mass concentration of the biomass standards was determined gravimetrically. Therefore, 5 mL of the standard was centrifuged and washed in deionized water two times using centrifugation (200× *g*, 10 min). Afterward, the resulting cell pellet was dried for 48 h at 80 °C and weighted after cooling in an exicator. Furthermore, the optical density of the biomass standards was determined.

In a further series of experiments, the conductivity in 5 different cell suspensions with a biomass ranging from 0 to 13.8 g L^−1^ was changed in a range from 2.81 to 6.68 mS cm^−1^ by adding sodium chloride (Sigma Aldrich, St. Louis, MO, USA) to the prepared cell suspension. The maximum sodium chloride concentration used was 50 mS mol L^−1^. The salt was added directly before the samples were stored on ice to reduce metabolic activity until measurement was performed.

### 2.4. Impedance Sensing

The outer electrodes of the measurment electrode system (MES), providing the AC current $\underline{I}$, were forming bows with a radius of 1.6 mm along the cwMBR sidewall covering an angle of 90° (Figure 2). The inner electrodes for probing the voltage $\underline{U}$ were parallel to the outer electrodes with a distance of 50 μm. The widths of all MES electrodes were 50 μm. The much smaller RES consisted of four parallel electrodes located on the cwMBR sidewall, pointing to the middle of the reactor. The distance between the inner electrodes was 80 μm, and that between the outer and the inner electrodes was 40 μm. All RES electrodes had a width of 30 μm and a length of 400 μm. The RES was covered with hydrogel to only measure the liquid without being influenced by the cells. The admittance as reciprocal of the impedance is given as

(2) $${\underline{Z}}^{-1}=R_{s}^{-1}+i\;\cdot \;2\pi f\;\cdot \;C_{s}$$

assuming that an Ohmic resistance $R_{s}$ is connected in parallel with a capacitance $C_{s}$, which is proportional to the permittivity at lower frequencies, $\epsilon_{l}$, both increasing with growing biomass. From here, the measured impedance spectra $C_{s}$ can be calculated as $C_{s}=Im\left({\underline{Z}}^{-1}\right)/2\pi f)$.

${\underline{Z}}^{-1}$ was measured with a potentiostat (Gamry Reference 600+, Gamry Instruments, Philadelphia, PA, USA). The electrodes were connected with gold-coated spring contacts to a PCB, which included two switches (G6K-2F-Y DC3, Omron, purchased from RS-Components GmbH, Frankfurt a.M., Germany), switching between MES and RES. The measurement was controlled using Gamry framework software (Version 7.8.6), activating the MES and RES with a potentiostatic controlled current at an AC voltage of 40 mV in a frequency range from 50 kHz to 5 MHz with 40 data points per decade.

The low resistance of the swollen hydrogel can lead to high currents. To avoid overload of the small current carrying outer RES electrodes, one outer was connected to one inner electrode, making the RES a 3-electrode system.

The EIS data were fitted based on a Matlab^®^ (The MathWork Inc., Natick, MA, USA) script [39], which was edited to match the purpose of this work. The fitting of a given equivalent circuit model (ECM) to the EIS data is based on the build-in function of Matlab^®^ fminsearch, looking for the minimum of the given problem, using the simplex method [40]. The original form of the script takes an ECM as input from the user and generates the transfer function from it. For this work, the transfer functions of the ECM for the RES and the MES were entered directly. In addition, the script was automated, and it processed several measurements in succession.

## 3. Results

### 3.1. cwMBR Fabrication

**Figure 3:**
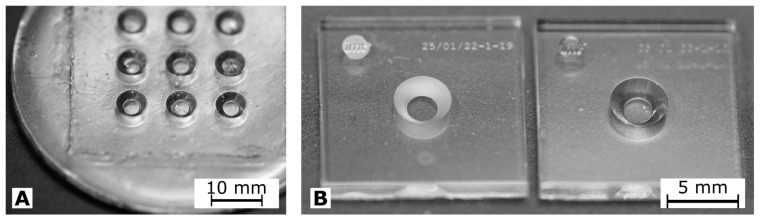
(**A**) Photograph of a cwMBR array manufactured using glass molding. (**B**) Photograph of a cwMBR manufactured using laser ablation with bonded bottom wafer before (left) and after thermal annealing at 760 °C (right).

**Figure 4:**
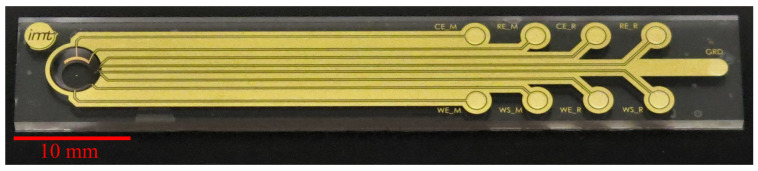
Photograph of the cwMBR-system with impedance sensor structures.

In Figure 3, photographs of cwMBRs manufactured by molding and by ablation are shown. Both manufacturing methods resulted in transparent systems. The systems made out of glassomer^®^(Glassomer GmbH) exhibit some waviness and small grooves. The waviness can be explained by the use of the very soft PDMS master. The grooves can be explained by particles on the PDMS, which quickly contaminate the master’s sticky surface. cwMBRs made by molding exhibit a roughness of $S_{a}$ = 0.12 μm. cwMBRs made by ablation initially exhibit a roughness $S_{a}$ of 0.32 μm measured by laser scanning microscopy (VK-X260K, Keyence, Osaka, Japan) which could be lowered to 0.07 μm by thermal annealing. Even though the glass molding technology can generate complex structures in one process, the obtained surface quality and planarity did not meet the requirements for lithographic electrode fabrication. Therefore, ablation and bonding were used to manufacture cwMBRs equipped with microelectrodes for biomass sensing. Seven cwMBR-systems with biomass sensing electrodes ($10\times 60\;mm^{2}$) as shown in Figure 4 could be located on one 4 –inch wafer. Four contacts were addressing the MES, four contacts were addressing the RES and one contact to ground was shielding the conductive paths.

### 3.2. 2PP Hydrogel

**Figure 5:**
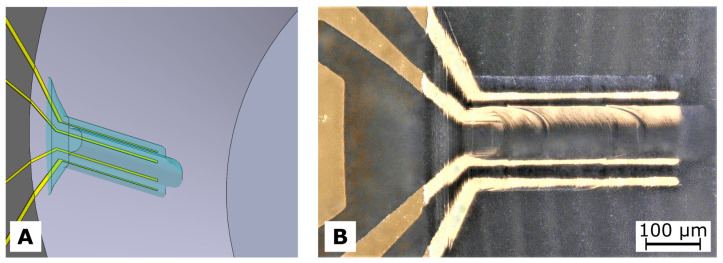
(**A**) Sketch of the RES covered with cross-linked hydrogel (cyan). (**B**) Micrograph of the RES covered with translucent swollen hydrogel inside the cwMBR filled with pure water. The image was taken using a digital microscope (VHX-5000, Keyence).

Prior to printing into the cwMBRs, the two-photon cross-linking of hydroxyethyl starch methacrylate hydrogel was extensively studied. Based on the findings presented in Appendix A.2, optimal printing parameters were determined to protect the RES: $v_{y}=1\;mm\;s$^−1^, Δz=5 μm, and Δx=1 μm. These parameters ensure high printing quality from the bottom (z=900 μm) to the top (z=100 μm) of the structure. As shown in Figure 5B, the hydrogel effectively covers all four electrodes of the RES.

### 3.3. Biomass Sensing

#### 3.3.1. 4-Probe Measurement

**Figure 6:**
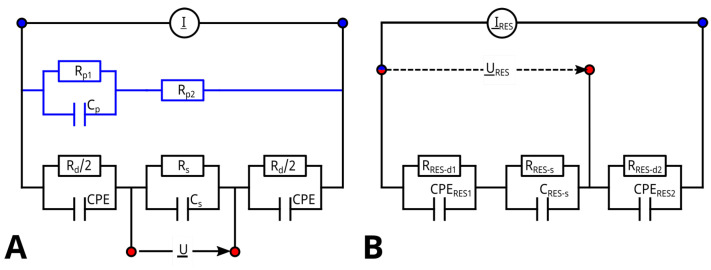
(**A**) ECM of the MES consisting of four electrodes. The outer electrodes are carrying the current $\underline{I}$ which is controlled by the potentiostatic voltage $\underline{U}$, measured at the inner electrodes. The element $R_{d}$ and CPE are representing electrode impedance, whereas $R_{s}$ and $C_{s}$ are representing the medium resistance and the cell capacity. The blue path with $R_{p1}$, $C_{p}$ and $R_{p2}$ represents a typically small parasitic branch. (**B**) ECM of the RES consisting of three electrodes. The potentiostatic voltage $\underline{U_{RES}}$ is measured with help of a reference electrode (red) and controls the current $\underline{I_{RES}}$. It consists of three parallel RC-circuits connected in series. The first ($R_{RES-d1}$ and $CPE_{RES1}$) models the electrode-based impedance. The second ($R_{RES-s}$ and $C_{RES-s}$) models the resistance and capacity of the medium soaked into the hydrogel. The third RC circuit models the impedance of the counter electrode but does not influence the measurement results.

The MES consisted of two outer electrodes connected to a current source ($\underline{I}$), which is controlled by the potentiostatic voltage ($\underline{U}$) at the inner electrodes. The measurement resulted in the complex impedance ${\underline{Z}}_{MES}\left(f\right)=\frac{\underline{U}}{\underline{I}}$ as a function of the frequency *f*. An ideal ECM of a 4-probe measurement is shown in Figure 6A. $\underline{I}$ is induced through the outer electrodes shown, represented by two RC circuits with a cumulated polarization resistance $R_{d}$ and two double-layer capacities, which are modeled with two constant phase elements (CPE) with an cumulated impedance of ${\underline{Z}}_{CPE}=\frac{1}{Y_{0}{\left(\omega j\right)}^{\alpha }}$. *U* is measured in parallel to the suspension resistance $R_{s}$ and the capacity of the suspension $C_{s}$, which depend on the medium used and on the amount of suspended cells. The measured impedance in an ideal 4-point measurement is independent of the electrode impedance, and $C_{s}$ can be obtained from the imaginary reciprocal impedance $Im({\underline{Z}}_{MES}^{-1})$. For frequencies far lower than the dispersion frequency of yeast cells at around 1.5 MHz, $C_{s}$ should not vary with frequency.

**Figure 7:**
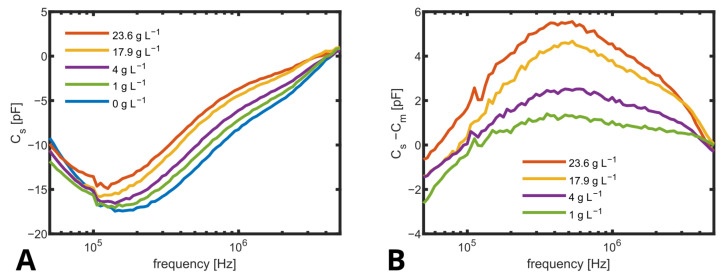
(**A**) Capacity spectra of biomass standards with different amounts of suspended yeast from 0 to 23.6 g L^−1^. $C_{s}$ was directly calculated as $C_{s}=2\pi f\;\cdot \;Im\left({\underline{Z}}^{-1}\right)$. Shown are the mean values of three different standards for each biomass. (**B**) $C_{s}-C_{m}$ of biomass standards with different amounts of suspended yeast from 0 to 23.6 g L^−1^, where $C_{m}$ is the spectra of the medium without biomass.

**Figure 8:**
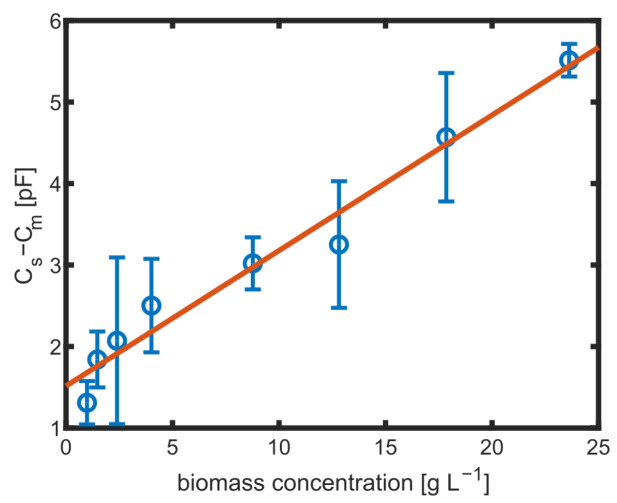
Change in capacity ($C_{s}-C_{m}$) at 0.5 MHz for different amounts of biomass with error bars showing the standard derivation of n=3 and a linear fit (red).

But it was found that $C_{s}$ determined using (2) based on measured impedance values as given in Figure 7A varied not only with concentrations of suspended yeast cells but also with frequencies even below 1.5 MHz. The typical course of β-dispersion (see also Figure 1) in yeast cells suggests a decreasing capacity between ≈0.5 and ≈10 MHz [41], which is also found in the processed capacity up to the limit of the measurement range (5 MHz). This reveals that ideal four-point measurement conditions were not given due to a parasitic current, as indicated in Figure 6A and discussed in the literature [13]. To compensate for variations in the media and to better represent the β-dispersion of the cells, the spectrum for $C_{s}$ obtained at 0 g L^−1^ ($C_{m}$) was subtracted from all other spectra (Figure 7B), and the capacity as obtained for 0.5 MHz was used as a measure for biomass concentration, as shown in Figure 8.

The linear fit is in good agreement with the measurement, but it does not cross the axis origin. This could be explained by the fact that only for the measurement without cells, the culture medium was fresh without any consumption by the cells. The linear fit has a slope of 0.14 pF L g^−1^. Even smaller biomass concentrations below 5 g L^−1^ could be distinguished.

**Figure 9:**
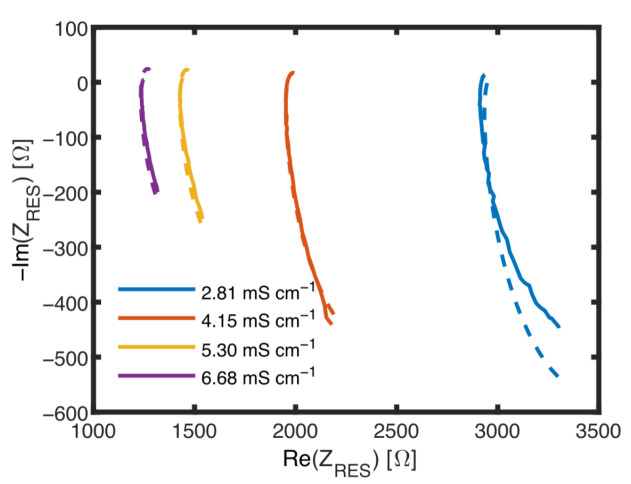
Nyquist plots of measurements with the MES at different conductivities with no biomass.

The measured current $\underline{I}$ is not completely flowing between the inner electrodes, because a parallel parasitic current splits off, which is influenced by the polarization resistance $R_{p1}$, a parasitic double-layer capacitance $C_{p1}$ and a parasitic medium resistance $R_{p2}$, as shown in Figure 6A. As a consequence, $C_{s}=\frac{Im(Z^{-1})}{2\pi f}$ used to determine biomass is no longer independent of changes in the conductivity of the medium. To analyze the parasitic pat, impedance data obtained without cells were fitted assuming the ECM including the parasitic elements. In the Nyquist plot shown in Figure 9, impedance data and fit results are shown for different media conductivities. Table 1 lists values obtained for all the elements.

In Figure 9, all curves are forming an unfinished semicircle representing the $R_{p1}$$C_{p1}$ circuit. The intersection with the abscissa correlates with $R_{s}$ and $R_{p2}$ and also with the conductivity of the suspension, whereas the bow below the abscissa and its minimum, always at 1 MHz, is affected by the $R_{d}$-CPE-Circuit and also the conductivity. This minimum is also, but not only, influenced by the capacity of the suspension $C_{s}$. To achieve consistent fit data as shown in Table 1, values of $Y_{0(MES)}$, $\alpha_{\left(MES\right)}$, $R_{p1}$ and $C_{p1}$ were in a second step fixed as averages of the initially found values, because these elements are practically not influenced by medium conductivity. $R_{d}$, $R_{c}$ and $R_{p2}$ depend on the conductivity of the medium. and it has been found that *R_p_*_1_ is directly proportional to $R_{s}$ with $R_{p}1=8.55\;\cdot \;R_{s}$. The influence of these elements is the dominating influences of $C_{s}$. It was expected that a change in the conductivity of the suspension would prevent the unambiguous determination of $C_{s}$. Therefore, the RES will be used to gain data about $R_{d}$ and $R_{s}$.

#### 3.3.2. Reference System

The ECM of the RES is shown in Figure 6B. It consists of similiar elements as the four-probe MES. The measured impedance includes the electrode impedance ($R_{RES-d1}$ and $CPE_{RES1}$) as well as the resistance and capacity of the medium soaked into the hydrogel ($R_{RES-s}$ and $C_{RES-s}$). It excludes the electrode impedance ($R_{RES-d2}$ and $CPE_{RES2}$) of the counterelectrode. A parasitic path must not be considered when measuring the voltage $U_{RES}$ resulting from the induced current $I_{RES}$ in a three-point configuration.

**Figure 10:**
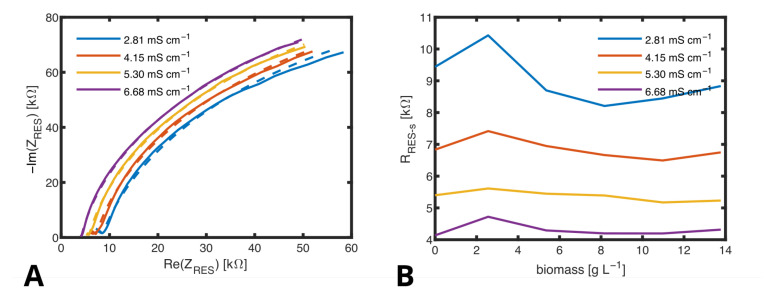
(**A**) Nyquist plots obtained from measurments with the RES in media with varied ionic concentrations and fitted curves (dashed lines). (**B**) $R_{RES-s}$, calculated by fitting several spectra measured in medium with biomass from 0 to 13.9 g L^−1^ and conductivities from 2.81 to 6.68 mS cm^−1^.

In Figure 10A, the course of the impedance obtained with the RES for different concentrations of sodium chloride is shown as a Nyquist plot. The frequency was varied in a range from 0.1 kHz to 100 kHz. When fitting the curves in the Nyquist plots to obtain the values for each component of the ECM, $C_{RES-s}$ and $R_{RES-d1}$ did practically not change with ionic concentration and could be fixed to $C_{RES-s}=79.6\;$pF and $R_{RES-d1}=19.9\;k\Omega$ in all fits. The curves were dominated by $CPE_{RES1}$ defining the semicircle in the Nyquist plot and $R_{RES-s}$ defining the starting point on the x-axes of this semicircle. The value of $Y_{0}$ does only slightly change in between the measurements in a range from 1.6 × 10^−8^ to 4.6 × 10^−8^ as the exponent α does from 0.84 to 0.99 and no correlation with the change of conductivity can be identified. Only $R_{RES-s}$ clearly decreases with ionic concentration from 10 kΩ at a low conductivity of σ=2.81 mS cm^−1^ to 10 kΩ at a high conductivity of σ=6.68 mS cm^−1^. Figure 10B shows the dependency of $R_{RES-s}$ on ionic concentration (conductivity of the medium). Since a dependency on biomass cannot be found, this figure proves that the hydrogel works as a cell filter and $R_{RES-s}$ determined by the RES is not influenced by cell density. The course of variations of $R_{RES-s}$ that were observed are similar for all measured conductivities. It can be assumed that these are due to slight deviations in preparation of the samples but not due to the change of biomass.

**Figure 11:**
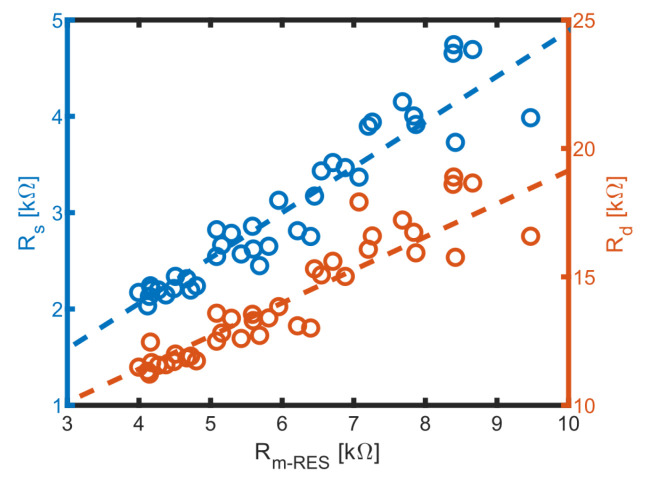
Correlation of medium related $R_{s-RES}$ to $R_{s}$ and $R_{d}$ and linear fits (dashed lines). The fits are resulting in $R_{d}=1.29R_{s-RES}+6.26\;k\Omega$ and $R_{p}=0.48R_{s-RES}+0.17\;k\Omega$.

Based on these results, the MES could be calibrated by correlating $R_{RES_{s}}$ with $R_{s}$ and $R_{p1}$ from the MES obtained in the same sample, as shown in Figure 11.

Linear correlations between $R_{s}$ and $R_{RES-s}$ as well as between $R_{d}$ and $R_{RES-s}$ were established. As conductivity decreases, uncertainties increase due to lower currents at potentiostatic voltages, which leads to reduced signal strength. These linear relationships allow us to derive $R_{d}$ and $R_{s}$ values from RES measurements when assessing biomass in media with unknown conductivity. Consequently, the only parameter that remains to be determined is $C_{s}$, which can be obtained by fitting the RES data.

**Figure 12:**
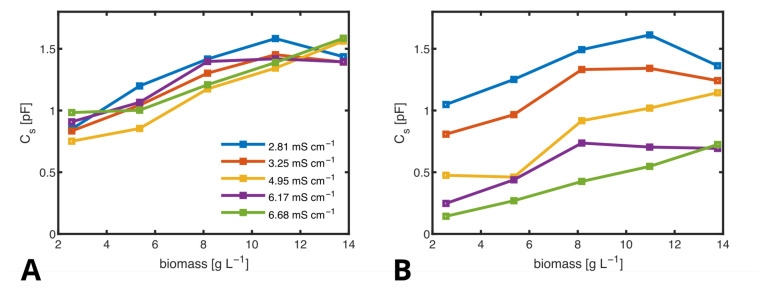
$C_{s}$ at different biomass concentrations, plotted against biomass, determined by fitting the ECMs using the RES (**A**) and without using the RES (**B**). Legend is the same for both figures.

By applying this method, we determined $C_{s}$ values from samples with biomass concentrations ranging from 2.6 to 13.8 g L^−1^ and conductivities between 2.81 and 6.68 mS cm^−1^, as shown in Figure 12A. For comparison, Figure 12B displays $C_{s}$ values obtained without correcting for $R_{d}$ and $R_{s}$ using the RES. Notably, in the latter case, the conductivity significantly influences the results. By employing the RES, we effectively suppress the impact of conductivity on $C_{s}$, ensuring that the measurement reflects biomass concentration rather than variations in conductivity.

Quantitatively, the linear correlation coefficient between $C_{s}$ and conductivity is −0.0036, indicating negligible influence, while the correlation between $C_{s}$ and biomass is 0.84, demonstrating strong sensitivity to biomass concentration. This distinction is crucial because the conductivity of the medium can change due to the metabolic products of the cells or the addition of ionic pharmaceutical ingredients.

In summary, our findings demonstrate that only by utilizing the RES can biomass be accurately measured, even in the presence of fluctuating conductivity. This capability is essential for reliable biomass monitoring in complex media where conductivity may vary over time.

## 4. Conclusions

In addition to the previously developed method of fs-laser direct writing in photosensitive glass, two less costly methods to manufacture the cwMBR were developed. Both the glass molding technology as well as the fs-laser ablation of glass are leading to transparent glass systems, which is important if using optical sensors inside the cwMBR. The ablation method is based on standard glass wafers, which can be further used for microtechnology processes, such as photolithography. The glass mold technology using glassomere^®^ offers higher flexibility regarding 3D shapes.

HES-MA was evaluated with regard to structurbility using 2PP with excellent results: It was possible to cross-link the hydrogel to form a freestanding structure by using printing speeds of up to 5 mm s^−1^. Furthermore, by adjusting the dose, the swelling can be influenced easily. To investigate this, a method was introduced, which enables us to observe the geometric swelling in the microscale. Maximum geometric swelling of 700% was achieved, which is in good agreement with values from the literature [34]. Cryo-SEM analysis supported the assumption that 2PP-HES-MA hinders yeast cells to enter the electrical field between the microelectrodes for impedance measurement due to small pore sizes in the submicrometer range. It could be shown that the HES-MA-covered RES was not influenced by biomass concentration, which allowed to compensate for the influence of the conductivity on the impedance spectra of the RES using the resulted values for fitting an ECM to the impedance spectra of the MES. Inside the MES ECM, a capacity ($C_{s}$) was identified, which did not correlate anymore with the conductivity but showed a strong correlation with the biomass, even when looking across samples with different conductivities.

Next, we will improve the design and the material of the sensor developed in this work. It was shown by Hofmann et al. [42] that the first has a big influence on the performance. The latter might help to reduce the artifacts occurring due to parasitic currents in this work. Furthermore—after the functionality has been proven—the use and applicability of the sensor developed here in a cultivation in a parallelised cwMBR-array will be demonstrated.

## Figures and Tables

**Table 1:** List of elements of the ECM shown in Figure 6A with their typical values, dependencies and meanings.

Element	Value	Dependency	Description
$R_{d}$	11–18 kΩ	positive correlation with conductivity	polarization resistance of the outer electrodes
$Y_{0}\left(MES\right)$	8.9 × 10^−11^	none, fixed	CPE, double-layer capacity of the outer electrodes
α(MES)	0.84	none, fixed	CPE exponent
$R_{s}$	2–5 kΩ	negative correlation with conductivity	medium resistance
$C_{s}$	0.19 pF	positive correlation with biomass expected, fixed on samples without biomass	capacity of the suspension
$R_{p1}$	2.3 kΩ	none, fixed	polarization resistance of the parasitic branch
$C_{p1}$	3.9 nF	none, fixed	double-layer capacity of the parasitic path
$R_{p2}$	17–42 kΩ	proportional to $R_{s}$, proportional factor based on an average over all samples measured without biomass	resistance of the medium in the parasitic path

**Figure A1:**
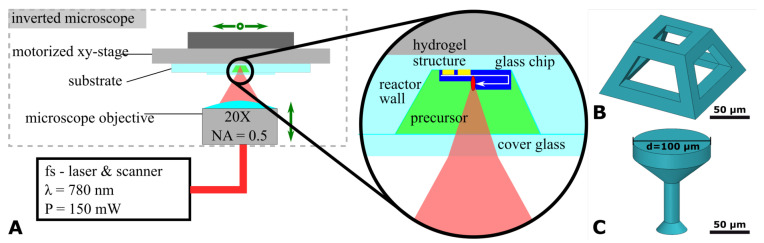
(**A**) Schematic representation of the 2PP setup with a zoom in on the laser beam positioning in the cwMBR. Also shown are CAD-models for pyramidal (**B**) and mushroom-shaped (**C**) test structures.

**Figure A2:**
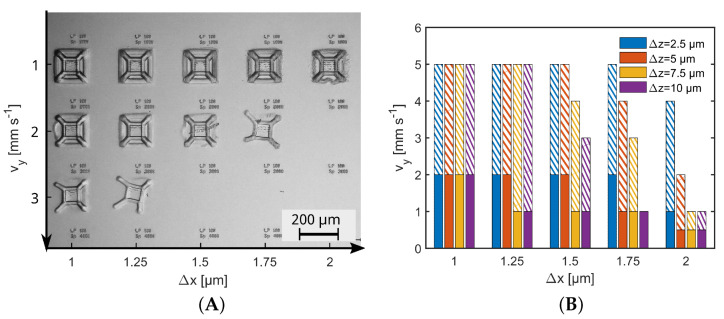
Parameter study for cross-linking HES-MA using 2PP. (**A**) Pyramid test structures as obtained with exemplary writing speeds $v_{y}$ and writing distances Δx. Δz was set to 5 μm. (**B**) Evaluation of all parameter tests, including a variation of Δz (different colors) The bars indicate the maximum writing speed at which stable and complete structures are still obtained. Different optical path through the precursor are indicated by filled (*z* = 700 μm) and hatched (*z* = 170 μm) bars.

**Figure A3:**
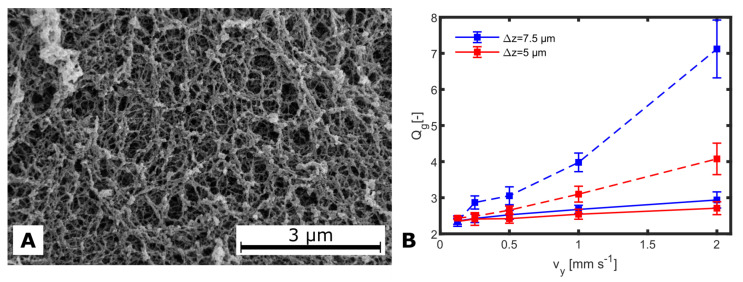
(**A**) Scanning electron micrograph of a 2PP-cross-linked hydrogel directly printed onto a sample carrier ($v_{y}=1\;mm\;s^{-1},\Delta z=5\;\mu m,z=900\;\mu m$ as determined by the geometry of the SEM sample carrier). (**B**) Geometric swelling $Q_{g}$ as a function of $v_{y}$ obtained with Δx=1 μm. Dashed lines are indicating z=700 μm, and solid lines z=170 μm. The color indicates two different values of Δz. The error bars are representing 3σ intervals.

## Data Availability

All research data will be made available upon request.

## Appendix A. Hydrogel Preperation and Testing

### Appendix A.1. Methods

HES-MA with a degree of substitution of 0.4 was synthesized as described earlier [34] and freeze-dried subsequently for convenient storage. Then, 30 wt% freeze-dried HES-MA was dissolved in filtered and deionized water before the solution was filtered and degassed. As a photo initiator, 0.9 wt% LAP (Sigma Aldrich, St. Louis, MO, USA) was added.

A 2PP system (Photonic Professional GT, Nanoscribe GmbH, Eggenstein-Leopoldshafen, Germany) equipped with a 20× objective (NA = 0.5, Zeiss, Jena, Germany) was used to locally cross-link the HES-MA. For printing inside the cwMBR, the precursor solution was directly filled into the cwMBR and then covered with a microscope cover glass (thickness 170 μm) as shown in Figure A1A. For initial studies, the cwMBR volumes were replaced by fs-laser-drilled holes in either 700 μm or 170 μm thick glass to provide optical paths through the precursor solution of different length. The reservoirs were closed with microscope cover glasses on which the structures were printed.

Two different test structures, a lattice pyramid with a diameter of 100 μm and a strength of the bars of 20 μm (Figure A1B) and a mushroom-like structure with a thin cylindrical stem of 20 μm diameter and a head of 100 μm diameter (Figure A1C), were printed. Depending on the 2PP parameters, complete and well-defined shapes, incomplete and unstable shapes or even the total absence of printed shapes were observed. The mushroom structure was used to examine the swelling of the HES-MA by measuring the diameter of the head after drying in ambient air for 12 h ($d_{d}$) and in the swollen immersed equilibrium state ($d_{q}$). The geometric swelling was calculated as

(A1) $$Q_{g}=\frac{d_{q}^{3}}{d_{d}^{3}}$$

$Q_{g}$ relates to the more often used gravimetric swelling $Q_{m}$ and the volumetric swelling [43] $Q_{V}$ given as

(A2) $$Q_{V}=\frac{Q_{g}}{r}=\frac{Q_{m}\rho_{h}}{\rho_{l}}+1$$

where $\rho_{l}$ is the density of water. The volume ratio *r* of the HES-MA in the dry state is <1 but not precisely known. Furthermore, the density of the HES-MA hydrogel cross-linked via 2PP $\rho_{h}$ can be estimated by comparing to similar hydrogels as $\rho \approx 1.42\;g\;{cm}^{3}$ [36].

For scanning electron microscopy (SEM), cubes with an edge length of 50 μm have been prepared and transferred to brass sample carriers filled with water and then shock frozen using a high-pressure freezer (Leica EM ICE, Leica Microsystems GmbH, Wetzlar, Germany). The samples were then freeze-etched for 10 min by sublimating some ice at −100 °C and then coated with platinum, using a desktop sputter system (Leica EM ACE600). The measurements were taken using a Helios G4 CX SEM (FEI Deutschland GmbH, Dreieich, Germany). During all process steps, the temperature was kept below −150 °C.

### Appendix A.2. Results

Laser power of 53 mW allowed high values for the scan speed $v_{y}$. Next to $v_{y}$, the lateral distance Δx between the scan lines and the vertical distance Δz between the layers filled with scan lines were also varied.

A parameter study was performed with two versions of the glass hole enabling a writing depth of z=700 μm, representing the deepest printing point inside the cwMBR and z=170 μm. Due to air/glass and glass/precursor interfaces, the focus became a little blurrier with increasing depth, leading to less focused energy deposition. Figure A2A shows the exemplary printing results of the parameter study, in which also the speed of line writing $v_{y}$ and the line distance Δx and Δz were varied. In Figure A2B, one can see decreasing light intensity with increasing values for *v_y_*, Δx and Δz, but *z* reached critical levels also, where instable or incomplete structures resulted. With Δx≥2 μm, there was no overlap between the scan lines and structures were not stable anymore. With Δx≤2 μm, stable and complete structures were obtained for all considered values of Δz; however, the writing speed had to be decreased to $v_{y}=0.5\;mm\;s$^−1^ to yield complete structures with Δx=2 μm. For Δx≤1.75 μm, complete and stable structures could be obtained with higher scan speeds up to $v_{y}=2\;mm\;s$^−1^. The printing depended strongly on *z*. The scan speed could be doubled or more than doubled for each single parameter set, when *z* was reduced from 700 μm to 170 μm where scan speeds up to a maximum of 5 mm/s were possible. That is is because in two-photon absorption, the averaged laser light intensity is not relevant but the local light intensities are.

A volumetric printing speed $v_{V}$ can be calculated as

(A3) $$v_{V}=v_{y}\;\cdot \;\Delta x\;\cdot \;\Delta z$$

In the parameter study, a maximum value of $v_{V}$ = $2\times 10^{-4}$ μm^3^ s^−1^ was reached at Δx=1 μm, Δz=10 μm, $v_{y}=2\;mm\;s$^−1^ and z=700 μm, meaning that a structure with a volume of *V* = $15\times 10^{6}$ μm^3^ (as required for the RES) could be printed in 13min.

Figure A3A shows a cryo-SEM image of a 2PP cross-linked hydrogel sample where the HES-MA exhibited pores in a range from 0.1– 0.5 μm in which yeast cells with a size of ≈5 μm [44] cannot enter or even pass. In contrast, the electrolytic components of the media will be able to diffuse through the HES-MA. In Figure A3B, the geometric swelling at different writing parameters is shown. Here, $v_{y}$ was changed from 0.125 to 2 mm s^−1^ for different *z* and Δz. A lower laser dose (high $v_{y}$ and Δz) results in higher $Q_{g}$. It covers a range from 2.3 to 7.1. Again, not only is the average dose important for cross-linking HES-MA but also the local rise above the threshold for two-photon absorption, which depends on the focusing ability of the optics. It seems that with increasing laser intensity, $Q_{g}$ asymptotically approaches a value of 2 which cannot be undershot, which is probably because the photoinitiator was completely consumed.
